# ALS-associated mutant FUS inhibits macroautophagy which is restored by overexpression of Rab1

**DOI:** 10.1038/cddiscovery.2015.30

**Published:** 2015-09-14

**Authors:** K Y Soo, J Sultana, AE King, RAK Atkinson, S T Warraich, V Sundaramoorthy, I Blair, M A Farg, J D Atkin

**Affiliations:** 1 Department of Biochemistry and Genetics, La Trobe Institute for Molecular Science, La Trobe University, Bundoora, Victoria, Australia; 2 Department of Biomedical Sciences, Faculty of Medicine and Health Sciences, Macquarie University, North Ryde, New South Wales, Australia; 3 Wicking Dementia Research and Education Centre, University of Tasmania, Hobart, Tasmania, Australia

## Abstract

Amyotrophic lateral sclerosis (ALS) is characterised by the formation of intracellular misfolded protein inclusions that form in motor neurons. Autophagy is the major degradation pathway for aggregate-prone proteins within lysosomes. Autophagy begins by the production of the omegasome, forming the autophagosome membrane, which then fuses with the lysosome. Mutations in fused in sarcoma (FUS) cause 5% of familial ALS cases and FUS-positive inclusions are also formed in sporadic ALS tissues. In this study, we demonstrate that the expression of ALS-associated mutant FUS impairs autophagy in neuronal cells. In mutant FUS-expressing neuronal cells, accumulation of ubiquitinated proteins and autophagy substrates p62 and NBR1 was detected, and formation of both the omegasome and autophagosome was inhibited in these cells. However, overexpression of Rab1 rescued these defects, suggesting that Rab1 is protective in ALS. The number of LC3-positive vesicles was also increased in motor neurons from the spinal cord of an ALS patient carrying a FUS (R521C) mutation compared with a control patient, providing additional evidence that autophagy is dysregulated in mutant FUS-associated ALS. This study provides further understanding of the intricate autophagy system and neurodegeneration in ALS.

## Introduction

Amyotrophic lateral sclerosis (ALS) is characterised by the degeneration of motor neurons in the brain, brainstem and spinal cord. Several proteins are genetically or pathologically linked to ALS, primarily superoxide dismutase 1 (SOD1), C9ORF72, Tar-DNA binding protein-43 kDa (TDP-43) and fused in sarcoma (FUS). FUS mutations have been described in human sporadic and familial ALS,^1,2^ and FUS-positive inclusions are present in affected tissues.^3,4^ FUS and TDP-43 are structurally and functionally very similar, with roles implicated in RNA processing and transport.

Autophagy is a major degradation pathway for intracytosolic aggregate-prone proteins, including those associated with neurodegeneration. Autophagy receptors p62 and NBR1 act as adaptors, linking the autophagy machinery to ubiquitinated protein substrates for degradation. When autophagy is inhibited, both p62 and NBR1 are upregulated.^5,6^ ATG9 is required for autophagosome biogenesis, and on induction of autophagy, it forms structures throughout the cytoplasm that overlap with LC3 puncta.^7–9^ The endoplasmic reticulum (ER) forms the omegasome, the precursor of the autophagosome,^10^ by double FYVE-containing protein 1 (DFCP1) binding to phosphatidylinositol-3-phosphate (PI(3)P) on the ER. Thus, cells labelled with DFCP1 allow for visualisation of early events in omegosome/autophagosome formation.^10^

Protein aggregation is a characteristic pathological hallmark of ALS. Intracellular inclusions containing misfolded proteins are formed in affected tissues, suggesting there is a cellular imbalance between generation and degradation of misfolded proteins. Autophagy is implicated in ALS and in the degradation of misfolded SOD1 and TDP-43.^11–13^ Autophagy is also dysregulated in cells expressing mutant TDP-43 and in SOD1^G93A^ transgenic mice.^13–15^ However, whether autophagy is impaired in cells expressing ALS-associated mutant FUS (mFUS) remains unclear.

Stress granules (SGs) are mRNA-protein containing aggregates induced during stress, and they accumulate in neurodegenerative diseases, including ALS. SGs are degraded by autophagy involving ubiquitin-selective chaperone valosin-containing protein, which is also mutated in familial ALS.^16^ Cytosolic mFUS is localised to SGs and FUS-positive cytosolic inclusions associate with SG proteins, including PABP, in brains of patients with ALS.^17,18^ Furthermore, mFUS-positive SGs co-localises with LC3-positive autophagosomes^19^ and autophagic substrate, p62 and LC3 co-localise with FUS-positive inclusions in sporadic ALS patients,^20^ linking RNA metabolism and polyubiquitinated protein aggregates to autophagy. Activation of autophagy with rapamycin also reduces the number of FUS-positive SGs.^19^ These findings indicate that the degradation of misfolded proteins by autophagy may be dysfunctional in FUS-linked ALS.

Rab1 proteins mediate all intracellular membrane trafficking events, including ER-Golgi trafficking and autophagosome formation.^21–23^ Increasing evidence now links ER-Golgi transport to autophagy,^21,24,25^ and we previously demonstrated that mFUS triggers ER stress in ALS.^26^ Here, we investigated whether mFUS inhibits autophagy, given the link to the ER. We found that the early stages of autophagy, the formation of the autophagosome and omegasome, were inhibited in cells expressing mFUS. However, overexpression of Rab1 restored autophagy and the recruitment of FUS to SGs in cells expressing mFUS. Hence, these data suggest that Rab1 activity is inhibited by mFUS in ALS.

## Results

### Overexpression of mFUS inhibits autophagy

**Figure 1:**
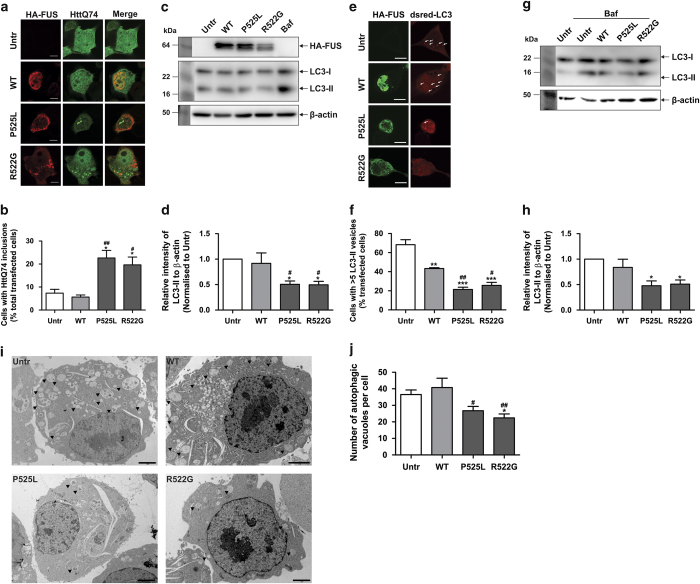
Overexpression of mFUS inhibits autophagy. (**a**) Representative images of Neuro2a cells co-transfected with HA-FUS (WT or mutant) and HttQ74-GFP constructs for 18 h. Cells were fixed and immunocytochemistry was performed using anti-HA antibodies, followed by confocal microscopy. Scale bar=10 *μ*m. (**b**) Quantification of the percentage of transfected cells bearing HttQ74 inclusions in (**a**), *n*=3. Untr represents untransfected cells. (**c**) Immunoblotting of soluble cell lysates from HA-FUS (WT or mutant) expressing Neuro2a cells using anti-HA and anti-LC3 antibodies. Blots were stripped and reprobed with *β*-actin as a loading control. Cells treated with bafilomycin A1 (Baf) were used as a control. (**d**) Quantification of the relative intensities of LC3-II from immunoblotting in (**c**), normalised to untransfected cells, *n*=5. (**e**) Representative images of Neuro2a cells co-transfected with HA-FUS (WT or mutant) and Dsred-LC3 for 18 h. White arrows indicate LC3-positive vesicles. Scale bar=10 *μ*m. (**f**) Quantification of cells in (**e**) containing >5 LC3-positive vesicles, *n*=3. (**g**) Neuro2a cells were transfected with HA-FUS (WT or mutant) for 18 h. Transfected cells were then treated with bafilomycin (100 nM)) for a further 6 h. Cell lysates were collected and subjected to immunoblotting using anti-LC3 antibodies. Blots were stripped and reprobed for *β*-actin as loading control. (**h**) Quantification of the relative intensities of LC3-II from immunoblotting in (**g**) from cells treated with Bafilomycin, normalised to untransfected cells, *n*=4. (**i**) Transmission electron microscopy images of Bafilomycin-treated Neuro2a cells expressing HA-FUS (WT or mutant). Arrow heads indicate autophagic vacuoles. Scale bars=2 *μ*m. (**j**) Quantification of the number of autophagic vacuoles per cell, from a total of 20 cells from each sample, *n*=2. Mean±S.E.M. One-way ANOVA with Tukey *post hoc* test. *P<0.05, **P<0.0001, ***P<0.00001 *versus* Untr, ^#^ *P*<0.05, ^##^ *P*<0.0001 *versus* WT.

First, we examined whether ALS-associated mFUS inhibits autophagy, by examining the accumulation of exogenously expressed human huntingtin with extended 74 CAG repeats (HttQ74) in Neuro2a cells. Neuro2a cells were co-transfected with GFP-HttQ74 and hemaglutinin (HA)-FUS tagged constructs for 18 h and cells were examined for the formation of GFP-HttQ74 inclusions, where an inclusion was defined as GFP-positive structures visible by light microscopy. In approximately 20% of cells expressing mFUS (P525L and R522G), GFP-HttQ74 inclusions were formed (Figures 1a and b). GFP-HttQ74 inclusions did not accumulate in cells expressing wild-type (WT) FUS or untransfected cells. These data imply that mFUS inhibits the clearance of HttQ74. As previous studies have demonstrated that HttQ74 is primarily degraded by autophagy,^27^ these data imply that autophagy/lysosomal function is impaired in cells expressing mFUS.

Autophagosome formation was next examined using immunoblotting and immunocytochemistry for LC3 following standardised guidelines for detecting autophagy.^28^ The relative intensities of LC3-II to *β*-actin (Figures 1c and d) and LC3-I (Supplementary Figure S1) using immunoblotting were analysed. In cells expressing mFUS (P525L and R522G), the relative intensity of LC3-II to *β*-actin and LC3-I was decreased compared with WTFUS-expressing cells or untransfected cells (Figures 1c and d and Supplementary Figure 1, *P*<0.05). Using immunocytochemistry followed by confocal microscopy, the number of LC3 vesicles/cell was also decreased in cells expressing mFUS compared with WT-expressing cells (*P*<0.0001 for P525L, *P*<0.05 for R522G) and untransfected cells (Figures 1e and f, *P*<0.00001). These data suggest that mFUS inhibits the formation of the autophagosome in Neuro2a cells. Expression of WTFUS also slightly but significantly decreased the number of LC3 vesicles/cell compared with untransfected cells (*P*<0.0001), although the levels of LC3-II by immunoblotting were unchanged in WTFUS-expressing cells compared with untransfected cells, implying that WTFUS also inhibits autophagosome formation, but to a lower extent than mFUS.

To confirm that the decreased levels of LC3-II were due to impaired autophagosome formation by inhibition of LC3-II synthesis, rather than increased degradation rates, we assayed LC3-II levels in the presence of bafilomycin-A1. Bafilomycin-A1 blocks LC3-II degradation by the autolysosomal pathway, and in cells treated with bafilomycin, LC3-II formation can be examined. The levels of LC3-II were decreased in mFUS-expressing cells that were treated with bafilomycin, compared with bafilomycin-treated untransfected or WT-expressing cells (*P*<0.05), confirming that autophagosome formation was impaired by mFUS (Figures 1g and h). We also examined cells treated with bafilomycin using transmission electron microscopy. Autophagosomes were identified by vacuoles containing ER membranes, mitochondria or ribosomes. Less autophagosomes were formed in cells expressing mFUS compared with WTFUS-expressing cells and untransfected cells treated with bafilomycin (Figures 1i and j, *P*<0.05). These data confirm that there were fewer autophagosomes in cells expressing mFUS because autophagosome formation was impaired.

### mFUS increases accumulation of autophagy substrates and ubiquitinated proteins in Neuro2a cells

**Figure 2:**
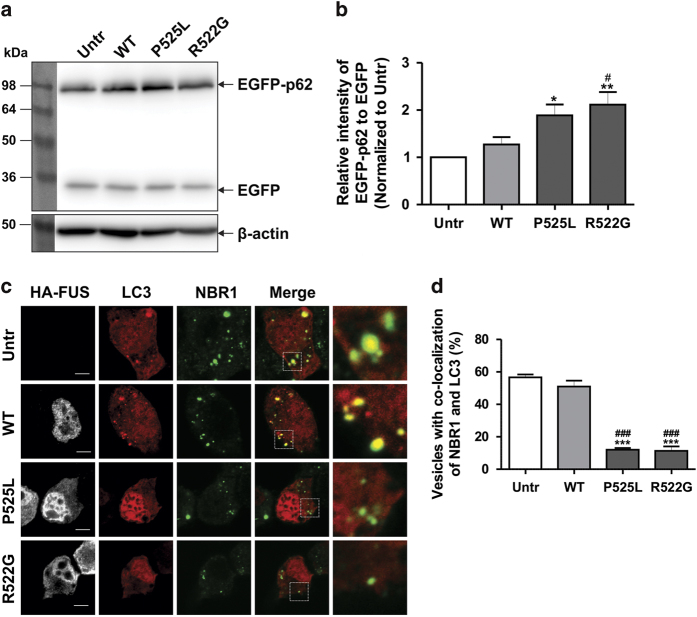
Overexpression of mFUS inhibits the clearance of ubiquitinylated proteins. (**a**) Effect of mFUS overexpression on the levels of p62. EGFP-p62 and EGFP (1.5 : 1) were co-transfected with HA-FUS (WT or mutant) in Neuro2a cells for 18 h. EGFP was used as a control for transfection efficiency of EGFP-p62. Cell lysates were collected and subjected to immunoblotting using anti-GFP antibodies. (**b**) Quantification of the relative intensities of EGFP-p62 from the immunoblots in (**a**), normalised to untransfected cells, *n*=5. (**c**) Neuro2a cells were co-transfected with HA-FUS (WT or mutant) and Dsred-LC3 for 18 h. Cells were then fixed and immunocytochemistry using anti-NBR1 antibodies was performed. Merge images and insets demonstrating co-localisation of LC3 and NBR1 are shown. Scale bar=10 *μ*m. (**d**) Quantification of the percentage of vesicles with co-localisation of LC3 and NBR1, *n*=3. Approximately 10 to 20 cells were scored in each experiments. Mean±S.E.M. One-way ANOVA with Tukey *post hoc* test. *P<0.05, ***P<0.00001 *versus* Untr, ^###^ *P*<0.00001 *versus* WT.

We next examined autophagy substrates, p62 and NBR1, which bind ubiquitin and accumulate when autophagy is inhibited.^29^ In cells expressing mFUS, increased levels of p62 were detected by immunoblotting compared with WTFUS or untransfected cells (Figures 2a and b, *P*<0.05). Similarly, in cells expressing mFUS, LC3 and NBR1 co-localised with fewer vesicles compared with WTFUS-expressing cells or untransfected cells (Figures 2c and d, *P*<0.00001). These data imply that less NBR1 is recruited to the autophagosome in mFUS expressing cells, suggesting that ubiquitinated proteins are removed less efficiently, providing further evidence that autophagy is impaired by mFUS.

### Formation of autolysosomes is inhibited in cells expressing mFUS

**Figure 3:**
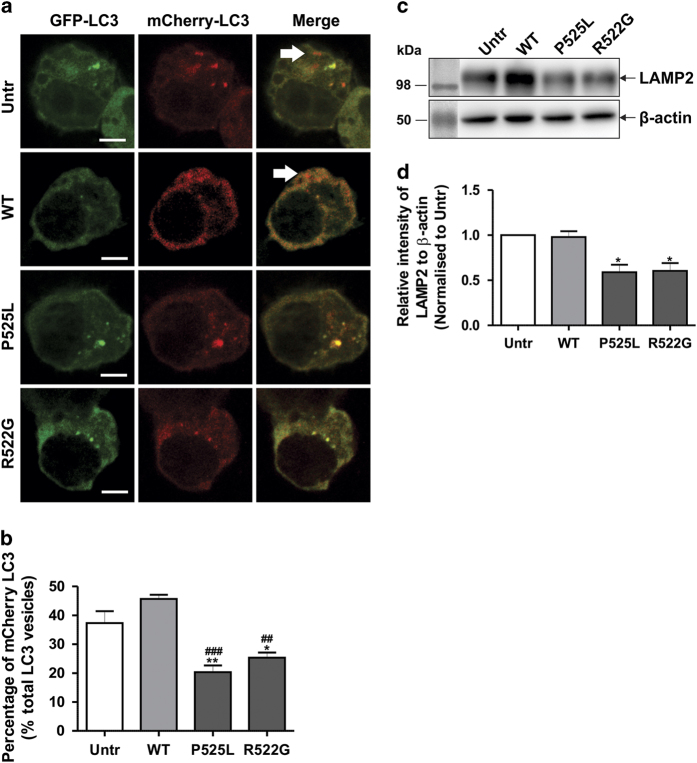
Formation of autolysosomes is inhibited in cells expressing mFUS. (**a**) Neuro2a cells were co-transfected with HA-FUS (WT or mutant) and mCherry-GFP-LC3 for 18 h before being examined using confocal microscopy. White arrows indicate mCherry-LC3 (indicating lysosomes). Scale bar=10 *μ*m. (**b**) Quantification of the percentage of vesicles with mCherry-LC3 from at least 10 cells of each sample, *n*=3. (**c**) SHSY5Y cells were co-transfected with HA-FUS (WT or mutant) and pcDNA-LAMP2C vectors for 18 h. Cell lysates were collected and subjected to immunoblotting using anti-LAMP2 antibodies. Blots were stripped and re-probed with *β*-actin as a loading control. (**d**) Quantification of the relative intensities of LAMP2 in (**c**), normalised to untransfected cells, *n*=2. Mean±S.E.M. One-way ANOVA with Tukey *post hoc* test. *P<0.05, **P<0.0001 *versus* Untr, ^##^ *P*<0.0001, ^###^ *P*<0.00001 *versus* WT.

We next examined whether the fusion of autophagosomes to lysosomes was impaired in mFUS-expressing cells. FUS proteins were co-expressed with double-tagged mCherry-GFP-LC3, which appears yellow (green merged with red) in non-acidic structures (autophagosomes and amiphisomes), and red in acidic autolysosomes, owing to quenching of GFP.^30^ In untransfected and WTFUS-expressing cells, 30–50% of LC3 vesicles were fluorescent red, indicating that lysosomes were formed. In contrast, significantly less red fluorescent LC3 vesicles were present in mFUS (P525L or R522G)-transfected cells (15–28%, Figures 3a and b, *P*<0.05), indicating that maturation of the autophagosome, and hence the formation of acidic autolysosomes, is inhibited in cells expressing mFUS. Similarly, the levels of lysosomal marker LAMP2 were significantly decreased in cells expressing mFUS compared with WTFUS or untransfected cells (Figures 3c and d, *P*<0.05). These findings indicate that fewer lysosomes were present in cells expressing mFUS.

### mFUS inhibits the co-localisation of autophagy protein ATG9 with LC3 vesicles

**Figure 4:**
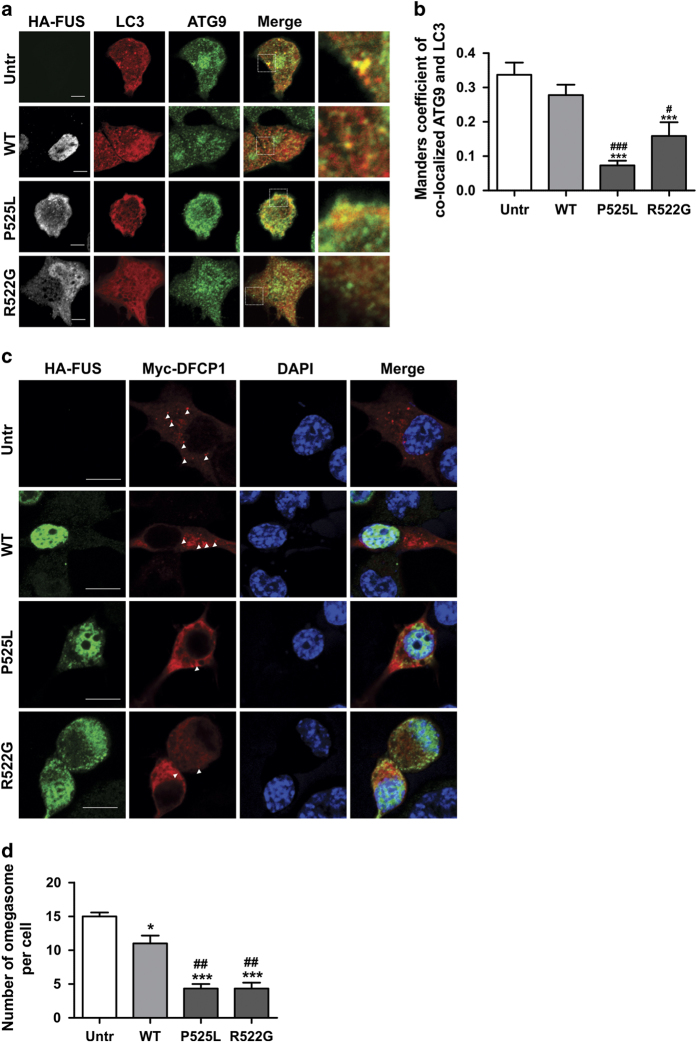
mFUS inhibits the recruitment of ATG9 to autophagosomes and the formation of omegasomes. (**a**) Neuro2a cells were co-transfected with HA-FUS (WT or mutant) and Dsred-LC3 for 18 h. Cells were fixed and immunocytochemistry using anti-ATG9 antibodies was performed. Merge images and inset demonstrating co-localisation between Dsred-LC3 and ATG9 is shown. Scale bar=5 *μ*m. (**b**) Quantification using Mander’s coefficient of the degree of co-localisation between ATG9 and LC3. A total of 20 cells were analysed from each sample, *n*=2. Mean±S.E.M. One-way ANOVA with Tukey *post hoc* test. ***P<0.00001 *versus* Untr, ^#^ *P*<0.005, ^###^ *P*<0.00001 *versus* WT. (**c**) Neuro2a cells were co-transfected with HA-FUS (WT or mutant) and myc-DFCP1 for 18 h. Cells were fixed and immunocytochemistry using anti-HA and anti-myc antibodies was performed. Cells were then counterstained with DAPI to identify the nucleus. White arrow heads indicate omegasome formation. Scale bar=10 *μ*m. (**d**) Quantification of the number of omegasomes formed per cell in (**c**). A total of 20 cells were analysed in each sample, *n*=3. Mean±S.E.M. One-way ANOVA with Tukey *post hoc* test. *P<0.05, ***P<0.00001 *versus* Untr, ^##^ *P*<0.0001 *versus* WT.

As ATG9 is recruited to LC3 vesicles (indicating autophagosomes) during autophagy, we next quantified the co-localisation between endogenous ATG9 and Dsred-LC3 in Neuro2a cells expressing FUS, using Mander’s coefficient. In untransfected cells and cells expressing WTFUS, ATG9 co-localised extensively with LC3-puncta, indicating that ATG9 is efficiently recruited to autophagosomes in these cells (Figures 4a and b). However, in cells expressing mFUS, significantly fewer ATG9-positive structures co-localised with LC3 vesicles (Figures 4a and b, *P*<0.05), indicating that translocation of ATG9 to autophagosomes is inhibited in cells expressing mFUS.

### mFUS inhibits formation of the omegasome

As we previously detected ER stress in mFUS-expressing cells,^26^ we next examined omegasome formation, by co-expression of mFUS with myc-tagged DFCP1. In cells expressing mFUS, the number of omegasomes present per cell was significantly reduced compared with WTFUS-expressing cells and untransfected cells (Figures 4c and d, *P*<0.001), implying that mFUS inhibits omegasome formation. Cells expressing WTFUS contained slightly, but significantly, fewer omegasomes/cell compared with untransfected cells (Figures 4c and d, *P*<0.05). These data suggest that mFUS inhibits omegasome formation, and hence the early stages of autophagosome formation.

### mFUS also inhibits autophagy in transfected mouse primary cortical neurons

**Figure 5:**
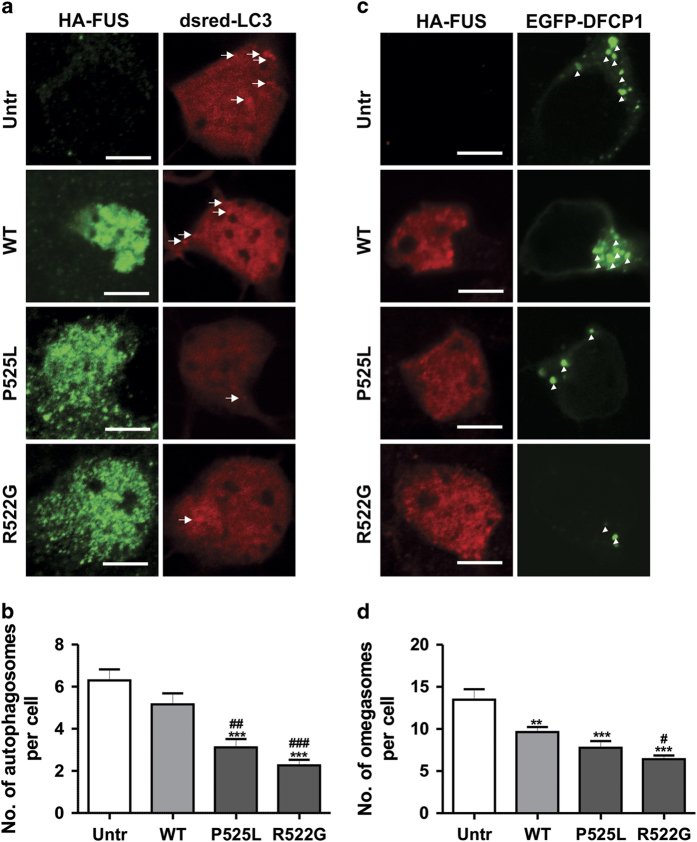
Less autophagosomes and omegasomes are formed in primary cortical neurons expressing mFUS. (**a**) Primary cortical neurons were co-transfected with HA-FUS (WT or mutant) and Dsred-LC3 for 18 h. Immunocytochemistry using anti-HA antibodies was then performed. White arrows indicate autophagosome formation. Scale bar=10 *μ*m. (**b**) Quantification of the number of autophagosomes formed per primary neuron in (**a**), *n*=3. (**c**) Primary cortical neurons were co-transfected with HA-FUS (WT or mutant) and EGFP-DFCP1 for 18 h. Immunocytochemistry using anti-HA antibodies was then performed. White arrow heads indicate omegasome formation. Scale bar=10 *μ*m. (**d**) Quantification of the number of omegasomes formed per primary neuron in (**c**), *n*=3. Mean±S.E.M. One-way ANOVA with Tukey *post hoc* test. *P<0.05, ***P<0.00001 *versus* Untr, ^#^ *P*<0.05, ^###^ *P*<0.00001 *versus* WT.

We next examined the inhibition of autophagy by mFUS in intact neurons to confirm these findings. Primary mouse cortical neurons were co-transfected with FUS and either Dsred-LC3 or GFP-DFCP1, to examine autophagosome and omegasome formation, respectively. In mFUS-expressing primary neurons, there was a significant decrease in the number of autophagosomes and omegasomes detected per cell, compared with cells expressing WTFUS or untransfected cells (Figure 5, *P*<0.05). In WTFUS-expressing cells, there was also a significant decrease in the number of autophagosomes, but not omegasomes, present per cell compared with untransfected cells (Figure 5d, *P*<0.0001). These data are consistent with the findings obtained in cell lines, and thus provide further evidence that mFUS inhibits the early stages of autophagy. Similarly, WTFUS inhibits autophagy, but to a lesser extent than mFUS.

### Rab1 rescues inhibition of autophagy induced by mFUS

**Figure 6:**
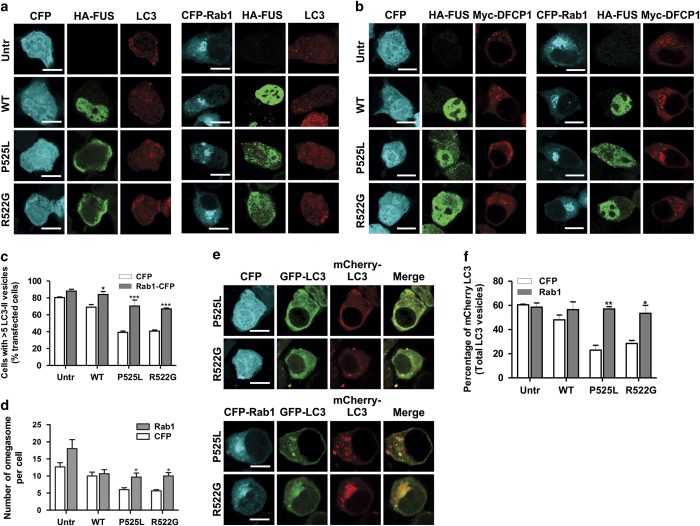
Rab1 overexpression restores autophagosome, omegasome and autolysosome formation in cells expressing mFUS. (**a**) Neuro2a cells were co-transfected with HA-FUS (WT or mutant), Dsred-LC3 and CFP-Rab1 vectors (or CFP empty vector) for 18 h. Scale bar=10 *μ*m. (**b**) Neuro2a cells were co-transfected with HA-FUS (WT or mutant), myc-DFCP1 and CFP-Rab1 (or CFP empty vector) vectors for 18 h. Scale bar=10 *μ*m. (**c**) Quantification of the percentage of cells in (**a**) with >5 LC3 vesicles per cell, *n*=3. (**d**) Quantification of the number of omegasomes per cell in (**b**), *n*=3. (**e**) Neuro2a cells were co-transfected with HA-FUS (WT or mutant), mCherry-GFP-LC3 and CFP-Rab1 vectors (or CFP empty vector) for 18 h. Scale bar=10 *μ*m. (**f**) Quantification of the percentage of vesicles with mCherry-LC3 signal in at least 10 cells from each sample, *n*=3. Mean±S.E.M. Two-paired Student *t*-test. *P<0.05, **P<0.0001, ***P<0.00001.

We next asked whether mFUS inhibits autophagy through Rab1-dependent mechanisms and whether overexpression of Rab1 can rescue autophagy inhibition. Rab1-tagged with CFP, or CFP only as a control, was overexpressed with HA-FUS (and DsRed-LC3) in Neuro2a cells. As expected, overexpression of CFP with mFUS P525L or R522G did not alter the formation of LC3 vesicles (Figures 6a and c) and significantly fewer mFUS-expressing cells contained >5 LC3 vesicles, compared with cells expressing WTFUS. However, co-expression of CFP-Rab1 with mFUS restored the number of cells with >5 LC3 vesicles to a similar proportion to those present in WTFUS-expressing cells and untransfected cells (Figures 6a and c). Similarly, overexpression of CFP-Rab1 restored the number of omegasomes detected per cell in mFUS P525L- or R522G-expressing cells, to levels similar to WTFUS-expressing or untransfected cells (Figures 6b and d). These data demonstrate that Rab1 is protective against inhibition of autophagy by mFUS. In contrast, overexpression of CFP did not restore omegasome formation in mFUS-expressing cells. We next examined whether Rab1 overexpression could restore autolysosome formation in cells expressing mFUS. Using double-tagged mCherry-GFP-LC3, Rab1 overexpression in cells expressing mFUS restored mCherry-LC3 vesicles (indicating autolysosomes), detected per cell to similar levels as in untransfected or WTFUS-expressing cells (Figure 6f). Hence, Rab1 restores autolysosome formation in cells expressing mFUS. Together, these findings therefore imply that the overexpression of Rab1 restores functional autophagy in cells expressing mFUS.

To confirm that the functional activity of Rab1 specifically restores autophagy, we co-expressed cells with FUS and two Rab1 mutants, inactive GDP-bound S25N, which cannot convert to its active GTP-bound form,^31^ or constitutively active Q70L, that remains GTP-bound.^32^ Cells were co-expressed with Dsred-LC3, FUS and Rab1S25N or Q70L (Supplementary Figure S2A). Unlike WTRab1, significantly fewer LC3 vesicles formed in cells co-expressing Rab1S25N and mFUS, untransfected and WTFUS-expressing cells (Supplementary Figure S2A, *P*<0.001). In contrast, control cells expressing CFP alone did not affect the number of LC3 vesicles present per cell. Similarly, the number of omegasomes per cell was also reduced in Rab1S25N-expressing cells for all cell populations examined, whereas control cells expressing CFP alone had no effect (Supplementary Figure S2A, *P*<0.05). Hence, inactive mutant Rab1S25N is not protective against inhibition of autophagy, unlike WTRab1. Furthermore, Rab1S25N also inhibits autophagy in control cell populations, consistent with previous studies.^22,33^ However, constitutively active mutant Rab1Q70L restored the levels of both autophagosomes and omegasomes in cells expressing mFUS, to levels similar to those present in WTFUS-expressing or untransfected cells (Supplementary Figure S2B, *P*<0.05). Taken together, these data indicate that mFUS dysregulates autophagy by inhibiting Rab1-dependent functions. It is important to note that the overexpression of mFUS did not affect the levels of Rab1 protein by immunoblotting (Supplementary Figure S3), implying that mFUS specifically inhibits Rab1 activity and does not affect the total protein levels.

### Overexpression of Rab1 is protective against recruitment of mFUS to SGs

**Figure 7:**
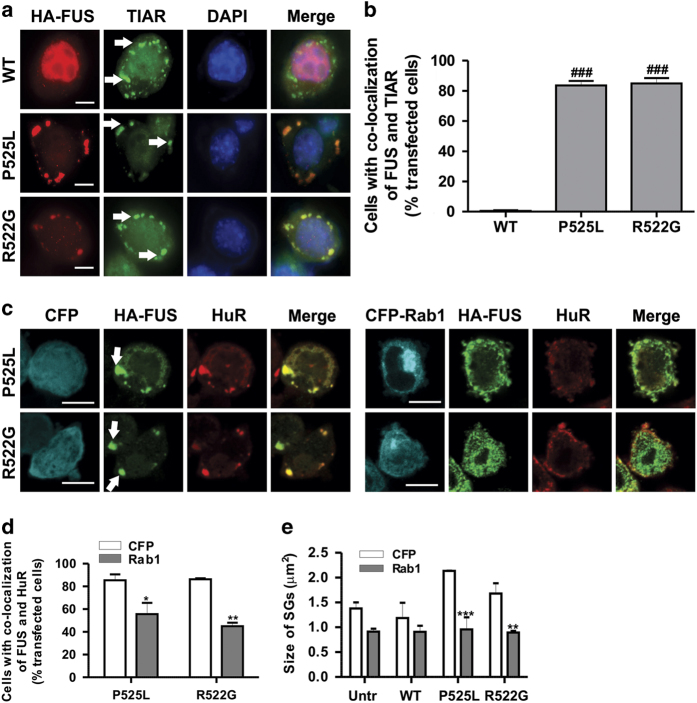
Rab1 overexpression inhibits the recruitment of mFUS to SGs and reduces the size of SGs formed. (**a**) Neuro2a cells were transfected with HA-FUS (WT or mutant) for 18 h. Cells were fixed and immunostained with anti-TIAR antibodies, and counterstained with DAPI to identify the nucleus. Merge images between HA, TIAR and DAPI are shown. White arrows indicate SGs. Scale bar=10 *μ*m. (**b**) Quantification of the % of cells displaying co-localisation between FUS and TIAR, indicating recruitment of FUS to SGs, *n*=3. (**c**) Neuro2a cells were co-transfected with HA-FUS (WT or mutant) and CFP-Rab1 vectors (or CFP empty vector) for 18 h. Cells were fixed and immunostained with anti-HuR antibodies and counterstained with DAPI to identify the nucleus. Merge images between HA, HuR and DAPI are shown. White arrows indicate SGs. Scale bar=10 *μ*m. (**d**) Quantification of cells displaying co-localisation between FUS and HuR, indicating recruitment of FUS to SGs, *n*=3. (**e**) Quantification of the size of each SG formed (*μ*m^2^). The size of SG was scored from at least 40 SGs in each sample, *n*=3. Mean±S.E.M. Two-paired Student *t*-test. *P<0.05, **P<0.0001, ***P<0.00001.

As Rab1 can restore autophagy in cells expressing mFUS and SGs are degraded by autophagy, we next investigated whether overexpression of Rab1 is protective against SG formation. Cells were treated with sodium arsenite to induce the formation of SGs, and immunocytochemistry for SG marker, TIAR, followed by counterstaining with DAPI was performed (Figure 7a). In untreated cells, no SGs were formed in any cell population, as expected (data not shown). In WTFUS-expressing cells treated with sodium arsenite, although SGs were formed in ~50% of cells, few SGs (<5%) co-localised with FUS, indicating that WTFUS was not recruited to SGs (Figures 7a and b). However, in cells expressing mFUS P525L or R522G, although similar numbers of SGs were formed as in WTFUS-expressing cells (50–60%), mFUS readily co-localised with SGs in 80% of these cells (*P*<0.0001, Figures 7a and b). Sodium arsenite induces oxidative stress, which is implicated in the normal aging process.^34^ Hence, oxidative stress induced by sodium arsenite, leads to the recruitment of mFUS to SGs in cells.

We next asked whether Rab1 overexpression inhibits the recruitment of mFUS to SGs. We co-expressed CFP or Rab1-CFP with FUS and performed immunocytochemistry for a second SG marker, HuR (Figure 7c). Both TIAR and HuR are widely used SG markers that bind to U-rich and AU-rich RNA, with high overall affinity for U-rich RNA.^35^ In control cells expressing mFUS and CFP, as expected, 80% of cells displayed FUS-positive SGs following sodium arsenite treatment (Figures 7c and d). Hence, these results confirm the findings obtained using HuR as a SG marker. However, in cells expressing mFUS and Rab1, mFUS was recruited to significantly fewer SGs compared with CFP-only expressing cells (two-fold decrease, *P*<0.05 for P525L and *P*<0.0001 for R522G, Figures 7c and d). Furthermore, the size of these SGs was significantly decreased in mFUS cells expressing Rab1 compared with CFP-only cells, from 1.5–2.2 *μ*m^2^ to 1.0 *μ*m^2^ (two to three-fold decrease, *P*<0.00001 for P525L and *P*<0.0001 for R522G, Figure 7e). These data therefore indicate that mFUS-positive SGs are regulated by Rab1-dependent autophagy in cells, and Rab1 expression inhibits recruitment of mFUS to SGs. Furthermore, sodium arsenite treatment also increases the number of LC3 vesicles in cells expressing mFUS (P525L or R522G) (Supplementary Figure S4, *P*<0.00001), but not in WTFUS-expressing or untransfected cells (Supplementary Figure S4).

### Increased numbers of LC3 vesicles are present in ALS patient carrying FUS mutation

**Figure 8:**
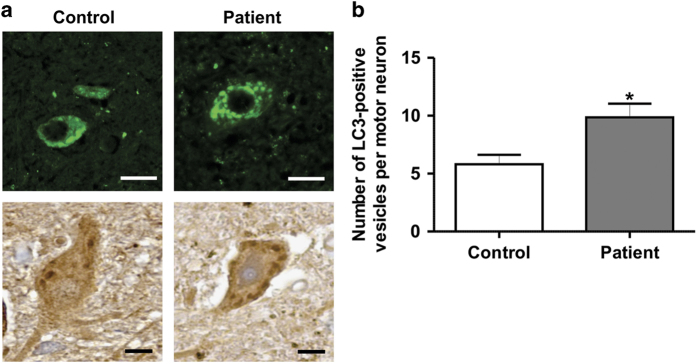
LC3-positive vesicles were increased in motor neurons from an ALS patient carrying R521C FUS mutation. (**a**) Immunohistochemistry of human post-mortem spinal cord sections (5 *μ*m) from a FUS mutation R521C ALS patient and a control case using an anti-LC3 antibody. Scale bar=40 *μ*m. (**b**) The number of LC3-positive vesicles was quantified from a total of 40 motor neurons per patient, *n*=40. Mean±S.E.M. One-way ANOVA with Tukey *post hoc* test. *P<0.05.

Finally, we examined ALS patient motor neurons for autophagy dysregulation. Immunohistochemical staining of post-mortem tissues from a control patient and a ALS-FTD patient bearing a FUS p.R521C mutation (previously described by Blair *et al.*,^3^ ALS53, individual IV:5) was performed using an anti-LC3 antibody (Figure 8a). There were significantly more LC3 vesicles present in the ALS mFUS-patient, compared with the control patient (Figure 8b, *P*<0.05), providing additional evidence that autophagy is dysregulated by mFUS in ALS.

## Discussion

It is currently unclear how mFUS triggers motor neuron degeneration in ALS. Dysfunction of autophagy is implicated in ALS and in disease models based on mutant TDP-43 and mutant SOD1.^13–15^ Here, we demonstrate that mFUS impairs early autophagy in neuronal cells. Furthermore, overexpression of Rab1 restores autophagy, prevents the recruitment of mFUS to SGs, and inhibits the formation of large SGs (>1 *μ*m^2^), implying a link between autophagy and SG formation.

We followed recommended guidelines for detecting autophagy,^28^ to determine that mFUS inhibits autophagosome formation in Neuro2a cells and primary cortical neurons. We detected fewer omegasomes and fewer LC3-positive autophagosomes in cells expressing mFUS compared with cells expressing WTFUS or untransfected cells. Furthermore, less ATG9 was recruited to autophagosomes and less lipidated LC3-II was present in mFUS-expressing cells. Autophagy substrates mutant huntingtin, p62 and NBR1, also accumulated in these cells. Although the omegasome is a precursor of the autophagosome, ATG9 is recruited to early autophagosomes prior to omegasome formation, and we detected little recruitment of ATG9 to LC3 vesicles. Hence, these data suggest that mFUS inhibits autophagy at an early step in autophagosome synthesis. The formation of autolysosomes in mFUS-expressing cells was also inhibited. This is most likely to be a direct result of fewer autophagosomes being present in mFUS-expressing cells, but we cannot rule out the possibility that mFUS also impacts upon autophagy degradation by the lysosomal pathway.

Autophagy dysfunction is increasingly implicated in the pathogenesis of ALS. Whereas some studies demonstrate that autophagy efficiently degrades ALS-linked mutant proteins in cell culture, other reports suggest that autophagy impairment contributes to disease pathogenesis.^36^ ALS patients show increases in autophagic markers,^37,38^ including p62, mTOR, Beclin-1 and ATG9. LC3-II and p62 levels were also increased in spinal cords of mouse models (transgenic SOD1^G93A^ ^39^ and SOD1^H46R^ ^40^ mice).^40^ Autophagy is also suppressed in the skeletal muscle of SOD1^G93A^ transgenic mice, which is related to enhanced apoptosis during ALS progression.^15^ These findings suggest that autophagy is induced to clear misfolded proteins in ALS. In this study, mFUS increased the levels of p62 and decreased the co-localisation between NBR1 and LC3 compared with WTFUS-expressing cells, implying that ubiquitinated proteins are removed less efficiently by autophagy in cells expressing mFUS. Furthermore, as the ubiquitin-proteasome system and autophagy are closely related, and excess p62 inhibits the clearance of ubiquitinated proteins destined for proteasomal degradation,^27,41^ this implies that inhibition of autophagy also compromises the ubiquitin-proteasome system.

Interestingly, in contrast, the number of LC3 vesicles was increased in motor neurons from a familial ALS patient with R521C FUS mutation. These data suggest that autophagosomes accumulate in cells expressing mFUS and they are consistent with dysfunctional autophagy in mFUS-ALS. However, the reason for the difference between the patient and cellular studies is not clear. One possibility is that although autophagosomes do not form as readily in mFUS-expressing cells, over the disease course in humans, this eventually inhibits functional autophagy, ultimately leading to autophagosome accumulation. Alternatively, the increase in autophagosomes in human patients is consistent with the fewer autolysosomes detected in cell culture. Finally, because mFUS inhibits autophagosome synthesis (Figures 1–5), it is possible that at disease end stage, the increase in LC3 vesicles in post-mortem tissues compared with controls is due to induction of non-canonical autophagy. Non-canonical autophagy can be triggered by the accumulation of reactive oxygen species, possibly from early autophagy deficits. It can bypass the stages involved in conventional autophagy, including omegasome formation, but it still depends on ATG5-LC3 and is independent of Beclin-1.^42,43^ It may therefore be speculated that although induction of non-canonical autophagy forms LC3 vesicles at disease end stage, the formation of autolysosomes is blocked, leading to the formation of large aggregates that trigger neurodegeneration.

Rab1 also restored the inhibitory effects of mFUS on omegasome, autophagosome and autolysosome formation. Similarly, Rab1 can rescue autophagy impairment induced by *α*-synuclein in Parkinson’s disease.^22^ It is therefore probable that mFUS antagonises Rab1-dependent formation of the autophagosome during autophagy.^21^ Autophagosome traffic on microtubules, and the Rab1 yeast homologue, Ypt1, mediates microtubule organisation and function.^44^ Hence, the overexpression of Rab1 may restore autophagy and fusion of the autophagosome to the lysosome by mediating microtubule function. Furthermore, analysis of RNA-binding targets of FUS using RIP-chip (RNA immunoprecipitation and microarray analysis) in NSC-34 cells revealed that FUS binds to Ras GTPase and Rab GTPase activator activity.^45^ This implies that FUS is an important regulator of GTPase activity. WT and mFUS binding has been mapped to Rab1 and other Rab transcripts,^46^ suggesting that Rab1 function is perturbed by mFUS. Similarly, ER and ubiquitin-proteasome-related genes were over-represented in transcripts that were uniquely bound by mFUS, thus linking mFUS to protein synthesis/degradation, and hence possibly autophagy.

Accumulation of p62 owing to deficits in autophagy may lead to aberrant induction of oxidative stress response genes,^47^ thus increasing the levels of reactive oxygen species, and inducing the formation of FUS-containing SGs. Here, we found that only mFUS recruits to SGs upon sodium arsenite treatment. This association was reinstated after Rab1 overexpression, suggesting that restoration of autophagy prevents the formation of mFUS-containing SGs. This indicates that efficient basal autophagy is required to prevent the accumulation of reactive oxygen species.^5^ Defects in autophagy could increase the levels of reactive oxygen species, leading to the sequestration of mFUS into SGs.^47^ Restoration of autophagy by Rab1 would therefore inhibit the recruitment of mFUS into SGs. Our data are consistent with a recent report showing that mFUS-positive SGs accumulate when autophagy is inhibited, but they disperse when autophagy is activated.^19^ Similarly, co-infection of adenoviral constructs encoding shRNA for proteins associated with the proteasome (PSMC1), autophagy (ATG5) and endosome (VPS24), with TDP-43 or FUS, enhances cytoplasmic TDP-43 or FUS aggregate formation in facial motor neurons. Hence, together these data imply that the autophagy pathway regulates FUS-positive SGs.^48^

This study therefore provides evidence that dysregulation of autophagy is a pathological mechanism in FUS-associated ALS. Thus, manipulation of the autophagic pathway and Rab1 may represent a possible therapeutic strategy for delaying disease progression in ALS-associated with FUS mutations.

## Materials and Methods

### Antibodies and chemicals

The antibodies used in this study included rabbit polyclonal antibody to HA (Sigma, Sydney, NSW, Australia; H6908) for immunocytochemistry (1 : 100), mouse monoclonal antibody to HA (Sigma; H3663) for immunocytochemistry (1 : 100) and immunoblotting (1 : 1000); rabbit polyclonal antibody to LC3 (Novus Biologicals, Littleton, CO, USA; NAB100-2220) for immunohistochemistry (1 : 100) and immunoblotting (1 : 1000), rabbit polyclonal antibody to Rab1 (Santa Cruz, Dallas, TX, USA; sc-28566) for immunoblotting (1 : 400), rabbit polyclonal antibody to NBR1 (Santa Cruz; sc-130380) for immunocytochemistry (1 : 50), rabbit monoclonal antibody to ATG9 (Abcam, Melbourne, VIC, Australia; ab108338) for immunocytochemistry (1 : 100), mouse monoclonal antibody to Myc (Santa Cruz; sc-40) for immunocytochemistry (1 : 50), mouse monoclonal antibody to HuR (Life Technologies, Mulgrave, VIC, Australia; 39-0600) for immunocytochemistry (1 : 100), mouse monoclonal antibody to TIAR (BD Transduction Laboratories, North Ryde, NSW, Australia; 610352) for immunocytochemistry (1 : 1000), mouse monoclonal antibody to LAMP2 (H4B4) (Abcam; ab25631) for immunoblotting (1 : 1000), mouse monoclonal antibody to *β*-actin (Sigma; A5441) for immunoblotting (1 : 4000), rabbit polyclonal antibody to GFP (Professor Mike Ryan’s laboratory) for immunoblotting (1 : 1000), Bafilomycin A1 was obtained from Sigma (B1793), sodium arsenite was obtained from Sigma (71287).

### DNA constructs

WTFUS and mFUS (P525L, R524S, R522G and R521G) constructs encoding HA-tagged human FUS at the N-terminus were a gift from Dr Dorothee Dormann, Ludwig Maximilians Universität München, Munich. EGFP-Q74 vector was a gift from David Rubinsztein (Addgene plasmid # 40262, Cambridge, MA, USA).^49^ mCherry-EGFP-LC3 vector was a gift from Jayanta Debnath (Addgene plasmid # 22418).^50^ Dsred-LC3 vector was a kind gift from Dr Yung-Feng Liao, Institute of Cellular and Organismic, Academia Sinica, Taiwan.^51^ Myc-DFCP1 and EGFP-DFCP1 vectors were kind gift from Dr Nicholas Ktistakis, Babraham Institute, United Kingdom. CFP-Rab1a vector was a kind gift from Professor Harald H. Sitte, Medical University Vienna. Mutant Rab1, S25N and Q70L, were generated by using Q5 Site-Directed Mutagenesis kit (New England Biolabs, Ipswich, MA, USA). p62-GFP vector was a kind gift from Professor Guanghui Wang, Soochow University College of Pharmaceutical Sciences, Suzhou. pcDNA-LAMP2C vector was a kind gift from Professor Tomohiro Kabuta, National Center of Neurology and Psychiatry; Kodaira, Tokyo, Japan.

### Cell culture and transfection

Mouse neuroblastoma Neuro2a cells or human neuroblastoma SHSY-5Y cells were maintained in DMEM supplemented with 2 mM L-glutamine, 10 mM HEPES buffer and 10% (v/v) fetal calf serum. Cells were maintained in a humidified 37 °C incubator with 5% CO_2_. Cells were transfected transiently with DNA constructs using Lipofectamine 2000 (Life Technologies; 11668) according to the manufacturer's instructions. For immunocytochemistry experiments, cells were seeded in 24-well plates containing 13-mm round coverslips.

Primary cortical neurons were isolated from mouse embryos at embryonic day 15.5. The top layers of both cortical hemispheres from embryos E15 mice were removed, meninges removed and trypsinised (0.0125% v/v, 5 min, 37 °C). Cortices were triturated in neuronal medium (Neurobasal containing 10% (v/v) fetal calf serum, 2% (v/v) B27 supplement, 0.5 mM L-glutamine, 25 *μ*M L-glutamic acid and 1% (v/v) antibiotic/antimycotic). Primary cortical neurons (at a density of 8×10^4^) were plated on poly-L-lysine-coated coverslips in neuronal medium.^52^ Transfections were performed as above at 48 h after plating.

### Immunocytochemistry and confocal imaging

Transfected Neuro2a cells were fixed in 4% paraformaldehyde in phosphate-buffered saline (PBS) for 10 min. Cells were washed three times with PBS after each step and permeabilised with 0.1% Triton-X-100, then blocked with 1% bovine serum albumin for at least 1 h at room temperature. Cells were then incubated with primary antibodies overnight and washed three times with PBS. Incubation with a suitable secondary antibody conjugated to Alexa Fluor secondary antibodies (Life Technologies) was at room temperature for 1 h. Nuclear morphology was also monitored by staining fixed cells with Hoechst (Life Technologies; 33342, 0.5 *μ*g/ml) for 5 min at room temperature and then cells were washed two times with PBS. Coverslips were mounted in fluorescence mounting medium (DAKO, North Sydney, NSW, Australia). Zeiss LSM 510 inverted confocal microscope equipped with a Confocor 3 system or Zeiss LSM 780 laser scanning microscope equipped with a PicoQuant FLIM system were used to acquire images (Carl Zeiss, Oberkochen, Germany). All images were obtained at room temperature. Images were collected using a ×100/1.4 Plan-Apochrome oil objective lens, green fluorescence was detected using an Argon laser, red fluorescence was detected using a DPSS laser, and far red fluorescence was detected using a HeNe laser. In dual-channel imaging, photomultiplier sensitivities and offsets were set to a level at which bleed-through effects from one channel to another were negligible. All images were processed using ImageJ and Zeiss software.

### Immunohistochemistry and immunofluorescence

Immunohistochemistry was performed on paraffin-embedded spinal cord tissues from individual IV-5 of family ALS53 (with R521C mutation) and another control individual without a neurological disorder. Patient tissue was kindly provided by P Blumbergs, J Manavis, and the NSW Brain Banks. Five-micrometre spinal cord sections were deparafiinised with xylene and rehydrated with a descending series of diluted ethanol and water. Antigens were retrieved by heating sections in 10 mM citrate buffer pH 6.0. Endogenous peroxidase activity was blocked with 3% hydrogen peroxide in methanol. Non-specific background was blocked with either 1.5% goat serum (Vector laboratories, Burlingame, CA, USA) for immunohistochemistry, or 1% bovine serum albumin (Sigma) for immunofluorescence. Sections were incubated in primary antibody, anti-LC3, at room temperature for 1 h. Alexa Fluor 488 (Life Technologies) was used as a secondary antibody. Biotinylated goat anti-rabbit IgG was used as secondary antibody for immunohistochemical staining. The avidin-biotin complex detection system (Vector Laboratories) with 3,3-diaminobenzidine as chromogen was used to detect the immunoreactive signal. The slides were counterstained with hematoxylin and a cover-slipped using DPX.

### Immunoblotting

Cells were lysed with Tris-NaCl (TN) buffer (50mM Tris-HCl, pH7.5, 150mM NaCl) containing 0.1% (w/v) SDS, with 1% (v/v) protease inhibitor mixture, for 10 min on ice. Lysates were clarified by centrifugation at 15 800×*g* for 10 min and the protein concentration of the cell lysates was determined using a bicinchoninic acid assay kit (Pierce Biotechnology, Rockford, IL, USA). Protein samples (20–30 *μ*g) were electrophoresed through 12% SDS-polyacrylamide gels and transferred to nitrocellulose membranes. Membranes were blocked with 5% skim milk/3% bovine serum albumin in TBS Tween 20 for 1 h then incubated with primary antibodies at 4 °C overnight. Membranes were incubated for 1 h at room temperature with secondary antibodies (HRP-conjugated goat anti-mouse or goat anti-rabbit) (Chemicon, Darmstadt, Germany), and were detected using enhanced chemiluminescence reagents (Roche, Kew, VIC, Australia). Quantification of blots was performed by ImageJ.

### Electron microscopy

Neuro2a cells were transfected with HA-FUS (WT, P525L and R522G) for 18 h. Cells were then treated with Bafilomycin A1 (100 nM) for a further 6 h. Cells were then trypsinised and fixed in 2% paraformaldehyde, 2.5% glutaraldehyde in 0.08 M Sorensen’s phosphate buffer/PBS for 30 min, then rinsed in PBS containing 5% sucrose. Post-fixation was carried out using 2% osmium tetroxide in PBS followed by dehydration through a graded series of alcohols, two acetone rinses and embedding in Spurrs resin. Sections 80-nm thick were cut with a diamond knife (Diatome, Nidau, Switzerland) on an Ultracul-S ultramicrotome (Leica, North Ryde, NSW, Australia) and contrasted with uranyl acetate and lead citrate. The sections were viewed under JEOL (Frenchs Forest, NSW, Australia) 1011 transmission electron microscope, equipped with Soft Imaging Systems MegaView III cooled CCD digital camera with iTEM imaging software/database.

### Statistical analysis

All data are expressed as the mean±standard error (S.E.M.) values and analysed for statistical significance by ANOVA followed by Tukey’s *post hoc* test or two-tailed Student *t*-test (GraphPad Prism, La Jolla, CA, USA). The differences were considered significant at *P*<0.05.

## Abbreviations

ALS: amyotrophic lateral sclerosis
FUS: fused in sarcoma
SOD1: superoxide dismutase 1
TDP-43: Tar-DNA binding protein of molecular weight 43 kDa
ER: endoplasmic reticulum
DFCP1: double FYVE-containing protein 1
PI(3)P: phosphatidylinositol-3-phosphate
WTFUS: wild-type FUS
mFUS: mutant FUS
SG: stress granule
HttQ74: huntingtin with extended 74 CAG repeats
S.E.M.: standard error of mean.
