# Supplementary material for: ALS-associated mutant FUS inhibits macroautophagy which is restored by overexpression of Rab1
Source: Cell Death Discov. 2015 Sep 14;1:15030–. doi: 10.1038/cddiscovery.2015.30 (PMC4979432; doi:10.1038/cddiscovery.2015.30)

Supplementary Materials

Supplementary Figure Legends

Supplementary figures

Supplementary Figure S1 – relates to Figure 1

Supplementary Figure S2 – relates to Figure 6

Supplementary Figure S3 – relates to Figure 6

Supplementary Figure S4 – relates to Figure 7

Supplementary Figure Legends

Supplementary Figure S1 Relative intensities of LC3-II to LC3-I from data presented in Figure 1C and D. n = 5. One-way ANOVA with tukey post-hoc test. *p<0.05 vs Untr, #p<0.05 vs WT.

Supplementary Figure S2 Inactive Rab1 mutant S25N cannot restore autophagosome and omegasome formation in cells expressing mFUS, unlike constitutively active Rab1 Q70L. (A) Neuro2a cells were co-transfected with HA-FUS (WT or mutant), dsred-LC3 or myc-DFCP1, and CFP-Rab1S25N (or CFP empty vector) for 18 h. Quantification of the percentage of cells with >5 LC3 vesicles per cell and the number of omegasomes present per cell is shown, n = 3. (B) Neuro2a cells were co-transfected with HA-FUS (WT or mutant), Dsred-LC3 or myc-DFCP1, and CFP-Rab1Q70L vectors (or CFP empty vector) for 18 h. Quantification of cells with >5 LC3 vesicles per cell and the number of omegasomes present per cell were shown. Scale bar = 10 µm, n = 3. Mean ± sem. Two-paired student t-test. *p<0.05, **p<0.0001, ***p<0.00001.

Supplementary Figure S3 Expression of endogeneous Rab1 is similar in WT and mFUS transfected cells. Neuro2a cells were transfected with HA-FUS (WT or mutant) for 72 h. Soluble cell lysates were collected and subjected to immunoblotting using anti-Rab1 antibodies. Blots were reprobed with β-actin as loading control.

Supplementary Figure S4 mFUS induces autophagy in Neuro2a cells upon sodium arsenite treatement. Neuro2a cells were co-transfected with HA-FUS (WT or mutant) and Dsred-LC3 for 18 h. Cells were then treated with 0.5 mM sodium arsenite for further 1 h. Cells were fixed and immunostained with anti-HA antibody. Transfected cells with >5 LC3 vesicles were quantified. n = 2. Mean ± sem. Two-way ANOVA with tukey post-hoc test. ***p<0.00001 vs non-treated.

Supplementary Figure 1

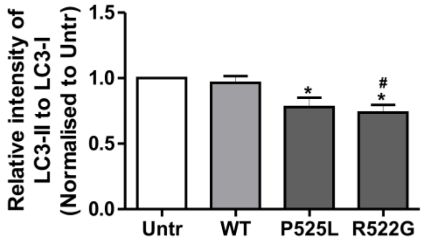

Supplementary Figure 2

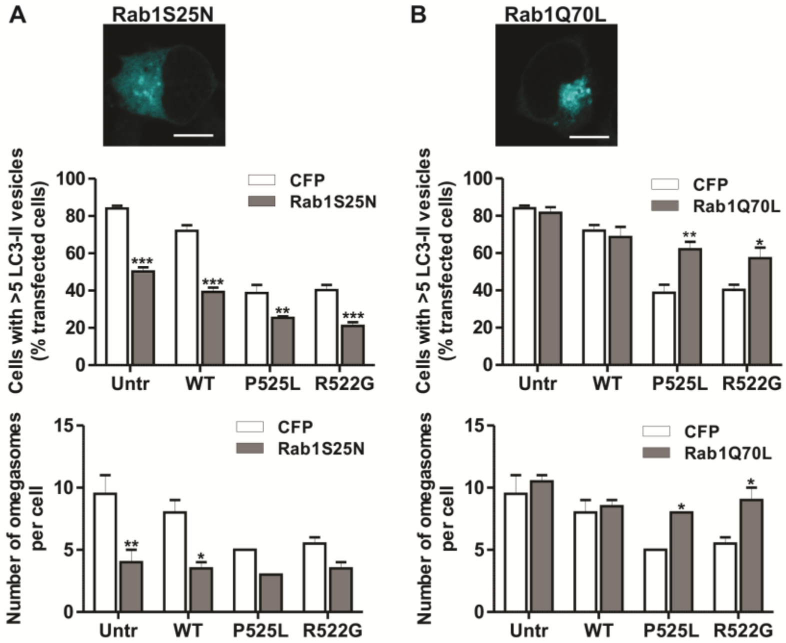

Supplementary Figure 3

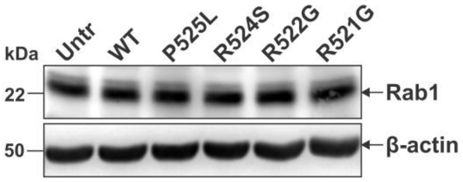

Supplementary Figure 4

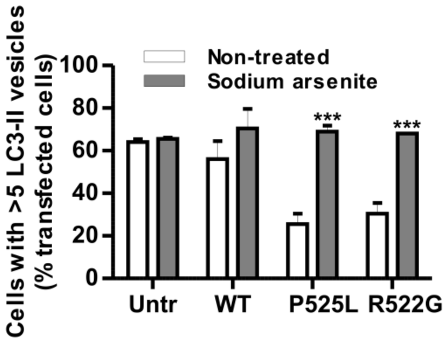

Supplement: Supplementary Information [file cddiscovery201530-s1.pdf]
